# Divergent effects of cerium oxide nanoparticles alone and in combination with cadmium on nutrient acquisition and the growth of maize (*Zea mays*)

**DOI:** 10.3389/fpls.2023.1151786

**Published:** 2023-03-30

**Authors:** Muhammad Ashar Ayub, Muhammad Zia ur Rehman, Hamaad Raza Ahmad, Cyren M. Rico, Ghulam Hassan Abbasi, Wajid Umar, Alan L. Wright, Muhammad Nadeem, John-Paul Fox, Lorenzo Rossi

**Affiliations:** ^1^ Institute of Soil and Environmental Sciences, University of Agriculture, Faisalabad, Punjab, Pakistan; ^2^ Institute of Agro-Industry and Environment, The Islamia University of Bahawalpur, Punjab, Pakistan; ^3^ Horticultural Sciences Department, University of Florida, Institute of Food and Agricultural Sciences, Indian River Research and Education Center, Fort Pierce, FL, United States; ^4^ Department of Chemistry and Biochemistry, Missouri State University, Springfield, MO, United States; ^5^ Institute of Environmental Science, Hungarian University of Agriculture and Life Sciences, Godollo, Hungary; ^6^ Soil, Water and Ecosystem Sciences Department, University of Florida, Institute of Food and Agriculture Sciences, Indian River Research and Education Centre, Fort Pierce, FL, United States

**Keywords:** engineered nanoparticles, cadmium stress, root barriers, root anatomy, nutrient uptake

## Abstract

**Introduction:**

The increasing use of cerium nanoparticles (CeO_2_-NPs) has made their influx in agroecosystems imminent through air and soil deposition or untreated wastewater irrigation. Another major pollutant associated with anthropogenic activities is Cd, which has adverse effects on plants, animals, and humans. The major source of the influx of Cd and Ce metals in the human food chain is contaminated food, making it an alarming issue; thus, there is a need to understand the factors that can reduce the potential damage of these heavy metals.

**Methods:**

The present investigation was conducted to evaluate the effect of CeO_2_-10-nm-NPs and Cd (alone and in combination) on *Zea mays* growth. A pot experiment (in sand) was conducted to check the effect of 0, 200, 400, 600, 1,000, and 2,000 mg of CeO_2_-10 nm-NPs/kg^-1^ dry sand alone and in combination with 0 and 0.5 mg Cd/kg^-1^ dry sand on maize seedlings grown in a partially controlled greenhouse environment, making a total of 12 treatments applied in four replicates under a factorial design. Maize seedling biomass, shoot and root growth, nutrient content, and root anatomy were measured.

**Results and discussion:**

The NPs were toxic to plant biomass (shoot and root dry weight), and growth at 2,000 ppm was the most toxic in Cd-0 sets. For Cd-0.5 sets, NPs applied at 1,000 ppm somewhat reverted Cd toxicity compared with the contaminated control (CC). Additionally, CeO_2_-NPs affected Cd translocation, and variable Ce uptake was observed in the presence of Cd compared with non-Cd applied sets. Furthermore, CeO_2_-NPs partially controlled the elemental content of roots and shoots (micronutrients such as B, Mn, Ni, Cu, Zn, Mo, and Fe and the elements Co and Si) and affected root anatomy.

## 1 Introduction

The rapidly increasing use of nanoparticles in diverse fields of electronics, pharmaceuticals, cosmetics, agriculture, and remediation technologies has created some serious concerns regarding their fate in the environment (Moraru et al., 2003; Ayub et al., 2022; Umar et al., 2022). The widespread application of nanoparticles has created many point and non-point sources of pollution, which can become a concern if they continue to be unchecked as metallic nanoparticles are a major class of these potential pollutants (Kurwadkar et al., 2015; Sohail et al., 2021). Agricultural land can become a major sink of these nanomaterials and very little information is known about their transformation in soil. Among metals, lanthanides are an important class of elements and cerium (Ce) is an abundant rare earth element (lanthanide) that has many proven beneficial roles (Yin et al., 2009; Shyam et al., 2012) and toxicities in plants (Zhang et al., 2015; Barrios et al., 2016), largely depending on source and concentration of application. Cerium is a non-essential element for plants but in lower concentrations has been proven to have beneficial effects for cowpea (Shyam and Aery, 2012) and rice (Ramírez-Olvera et al., 2018). However, at higher concentrations, it has shown toxicity in maize (Ayub et al., 2023). Cerium NPs are important as they are used in energy storage, polishing, personal care, cosmetics, biomedical industries, and as catalysts, and their market is projected to reach $2.1156 billion by 2030 (Allied Market Research Report A01390, 2021). As cerium and its nanoparticles are widely used in various industrial activities (Dahle and Arai, 2015; Rajeshkumar and Naik, 2018; Ayub et al., 2019), it has become an attractive topic of discussion due to the increasing risk of an influx into the human food chain *via* agriculture. CeO_2_-NPs have been categorized as a top 13 engineered nanomaterial (Organization for Economic Cooperation and Development, 2008) and have physical and chemical properties that make them suitable for a wide range of applications due to their crystal lattice oxygen chemistry (Rzigalinski et al., 2006; Korsvik et al., 2007; Jiao et al., 2012; Dowding et al., 2013).

A variety of reported effects have been reported for the application of CeO_2_-NPs in plants, ranging from nutrition management (Peralta-Videa et al., 2014; Adisa et al., 2019), disease and growth management (Servin et al., 2015), and abiotic stress management (Rossi et al., 2016) to toxicity at higher concentrations (Iftikhar et al., 2020). The complex surface chemistry, fate in soil, and soil-NP-plant interaction have caused a significant divergence in the results obtained from Ce-NP application in plants, resulting in beneficial (Rossi et al., 2016), toxic (Morales et al., 2013; Iftikhar et al., 2020), or minimal effects (Corral-Diaz et al., 2014). CeO_2_-NP size (Lizzi et al., 2020; Deshpande et al., 2005; Dinesha et al., 2021), surface chemistry (Liu et al., 2019), contact time (Zhao et al., 2012), and application dose (Singh et al., 2019) can alter the effect of cerium NPs in plants (Hussain et al., 2019). The soil processes associated with cerium NP application, such as soil aggregation, dissolution, and sorption, control the fate of these nanoparticles in soil (Batley and McLaughlin, 2010). The application of cerium NPs (8 ± 1 nm) can improve the antioxidant potential in *Raphanus sativus* L. applied at 250 mg kg^-1^ (Corral-Diaz et al., 2014) and alter the growth (applied at 0−800 mg kg^-1^) of *Helianthus annuus* L. (Tassi et al., 2017), but has resulted in toxicity at higher concentrations due to a deterioration in the nutritional value in *Oryza sativa* (Rico et al., 2013a; Rico et al., 2013b) and oxidative stress in *Zea mays* (Zhao et al., 2012). Cadmium (Cd) is another pollutant that is added to agroecosystems through various anthropogenic activities and has well reported adverse effects on plants (Haider et al., 2021) and humans (Kumar and Sharma, 2019). The co-existence of CeO_2_-NPs and Cd in soil can lead to variable effects in plants depending on NP source, dose, and exposure duration (Rossi et al., 2018; Sharifan et al., 2020; Liu et al., 2021).

Maize (*Zea mays*) is an important cereal crop, and our previous work has shown that pollutants such as CeO_2_-NPs and Cd can alter its morphology (Fox et al., 2020). Divergent effects of Ce sources exist (Ayub et al., 2023) but knowledge about the effect of high concentrations of very fine CeO_2_-NPs (10 nm) alone and in combination with Cd in maize is lacking. With this in mind, the present investigation was conducted to evaluate the effects of CeO_2_-10 nm NPs applied at 0, 200, 400, 600, 1,000, and 2,000 ppm in sand alone and in combination with Cd (0 and 0.5 mg kg^-1^) on the growth, nutritional distribution, and root anatomy of maize seedlings.

## 2 Material and methods

### 2.1 Material collections, preparation, and characterization

The cerium oxide nanoparticles (CeO_2_-NPs 10 nm powder) were purchased from U.S. Research Nanomaterials, Inc. (Houston, TX, USA) with 99.99% purity and in 100 g packing. Cadmium salt (Cadmium sulphate CdSO_4_), nitric acid (TraceMetal Grade, 67-70% HNO_3_), and H_2_O_2_ (JT Baker Hydrogen Peroxide, 30% ULTREX II Ultrapure Reagent) were purchased from Fisher Scientific Int. (Pittsburgh, PA, USA). Sakrete sand (Atlanta, GA, USA) was used and Hoagland salt mixture (Hoagland Complete Medium) from planmedia.com was used as the nutrient source. The 50 g of sand and 5 g of nanoparticles were sent to the Nanoscale Research Facility (NRF) at the University of Florida Gainesville Campus for SEM (FEI NOVA 430 Nano SEM; voltage 5kV; spot size of 3.0; and a working distance of 4.6 mm magnification from 200 to 400 kX) and energy disruptive X-ray spectroscopy (10 kEV; data collected for 50 s for each sample at a working distance of 5 mm and a spot size of 4.5). Plastic cups (volume of 500 ml) were filled with 600 g of sand and spiked with 0, 200, 400, 600, 1,000, and 2,000 mg of CeO_2_-NPs per kg dry sand along with 0 or 0.5 mg kg^-1^ of Cd. There was a total of 12 treatment sets applied in 4 replicates under a two-way factorial design. The treatment sets were prepared at the Indian River Research and Education Centre (IRREC), University of Florida (UF), Fort Pierce, FL, USA on July 24th, 2021, *via* solution (Cd) and dry mixing (CeO_2_-NPs).

### 2.2 Plant growth and propagation

Hybrid Bicolor Synergistic Corn seeds (*Zea mays*) were purchased from Johnny’s Selected Seeds (Winslow, ME, USA) and soaked (for surface sanitized) in 10% commercial bleach solution prepared in distilled water for 5 min, followed by triple washes with plenty of distilled water. From these surface sanitized seeds, five healthy seeds were sown in pots (pre-irrigated and pretreated) on July 28th, 2021, and after germination on July 31st, only one healthy seedling was retained in each pot and irrigated with 10 ml of 50% Hoagland solution for the next few days. From August 4th, pots were irrigated with 15 ml of 50% Hoagland solution, followed by 100% Hoagland solution irrigations (uniformly in each pot). After attaining a significant height, the seedlings were harvested on August 26th and processed further for root, shoot growth, and biomass data recording.

### 2.3 Growth data recording

The plants were harvested treatment wise, and roots were separated from the shoot and washed with distilled water (first in the greenhouse and then in the lab) to remove any residue. Roots were scanned with an EPSON Perfection V800 photo scanner and images were analyzed using WinRHIZO pro software to generate root growth data, including growth parameters (number of tips, number of forks, number of crosses, root length per unit volume of sand [cm cm^-3^], average root diameter [cm], total root length [cm], total root surface area [cm^2^], total root projected area [cm^3^] and total root volume [cm^3^]). Additionally, the fresh weights of the roots were recorded. Subsequently, the two or three best developed and uniform root tips from each root were cut and placed in 15 ml of methanol (in Falcon tubes) and kept at 4°C for the next few days while the florescence root scanning setup was prepared (the difference in root weights pre and post tip sampling was recorded for later use in weight correction). Following this, shoot fresh weight, height, and diameter were recorded using a digital balance, scale, and caliper (Neiko 6” Stainless Steel Digital Caliper, Neiko Tools USA, China), and all samples were labeled and stored in paper bags and placed in the lab overnight. The next day, the samples stored in the paper bags were placed in an oven at 65°C for 5 days until a constant dry weight was achieved.

### 2.4 Elemental analysis

The wet acid digestion method (with 70% HNO_3_ and 30% H_2_O_2_) was used, for which a known mass of plant tissue was placed in digestion tubes and mixed with 10 ml of nitric acid (TraceMetal Grade, 67-70% HNO_3_, Fisher Chemical) and covered with glass funnels to assist the collection of back flow. The samples were kept overnight in a fume hood at the Soil and Water Science Laboratory, Indian River Research and Education Center (IRREC). The next day, the digestion tubes were adjusted on a temperature-controlled Digestion System 40 (model Tecator Digestion System) 1016 digester placed in a fume hood and the temperature was gradually raised to 75°C, which was maintained 1 h. Thereafter, a temperature of 95°C was maintained for 3 h and then samples were cooled to room temperature at which 2 ml of 30% H_2_O_2_ solution was added; the temperature was subsequently increased to 75°C to assist in the complete digestion. The digestates were diluted to 50 ml in the same tubes, then filtered through Whatman 42 filter paper and diluted again 10 times to achieve a net 2% acid content for analysis by inductively coupled plasma-mass spectrometry (ICP-MS). The elemental analysis was performed at the Chemistry and Biochemistry Department, Missouri State University using an ICP-MS Agilent 7900 equipped with a SP4 autosampler. The acquired concentration of all elements (ppm and ppb) was converted to mg kg^-1^ dry shoot mass using the following formula:


$$\begin{array}{l} {\text{Element Concentration in Tissue (mg kg}}^{-1})\\ =\frac{\left(ICPgivenconcentrationinppb\right)}{massoftissueusedfordigestion}\times \frac{50(finalvolummade)}{1000(conversionfactorppbtoppm)}\times 10(Dillution\;to\;achieve\;2\%\;acidlevel) \end{array}$$

The sand used in our experiment was play sand, of which 1 g was added to 20 ml of distilled water, and the solution elemental concentration was determined to estimate the potential of sand to release elements and nutrients under investigation.

### 2.5 Qualitative and quantitative identification of the root suberin lamella barrier

The tips selected from the roots of each maize plant were stained with fresh 0.01% w/v Fluorol Yellow solution (prepared in lactic acid) at 70 °C for 30 min in the dark, followed by counter staining with 0.5% w/v solution of Aniline Blue, as prescribed by Lux et al. (2005). After the first staining, tips were washed three times for 5 min each time. After the second staining, tips were washed three times for 10 min each time. Once stained, tips were mounted on labeled glass slides and visualized under a Leica DM 1000 LED (Wetzlar, Germany) florescence microscope equipped with a UV chamber, filter, and ImageJ (NIH, Bethesda MD) software. A digital caliper and ImageJ software were used to quantify the total length of suberin barrier formation from the root tip.

### 2.6 Statistical analysis

Twot-way ANOVA was used for the statistical classification of factors (Ce NPs and Cd) and treatments. Statistix version 10 was used for the statistical analysis and a correlation tree map was made using R (https://www.r-project.org/).

## 3 Results

### 3.1 Material characterization

**Figure 1:**
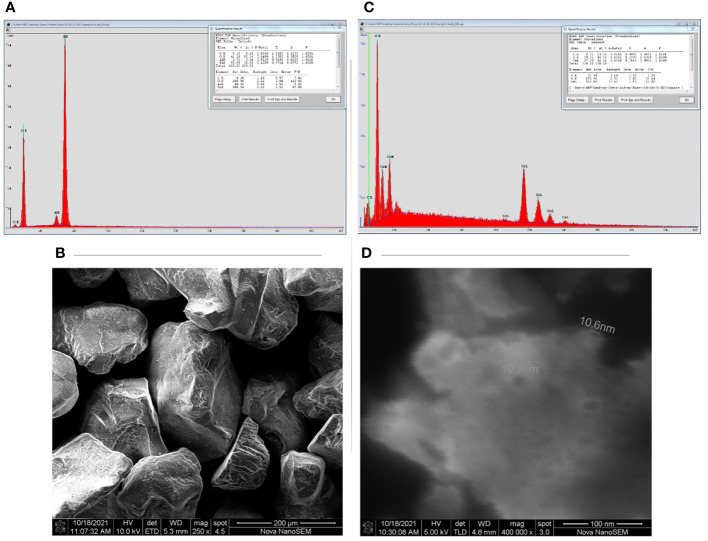
Scanning electron microscope images (SEM) and energy disruptive X-ray (EDX) peaks of sand and cerium oxide nanoparticles (CeO_2_ NPs): **(A)** Sand EDX peaks, **(B)** Sand SEM, **(C)** CeO_2_-NPs EDX, **(D)** CeO_2_-NPs SEM.

The sand used in our study has shown the potential to release significant amounts of elements under investigation given as mean µg kg^-1^ dry sand ± SD. The elemental concentrations were B (27.55 ± 2.41), Mn (24.45 ± 1 7.82), Ni (9.57 ± 4.97), Cu (112.84 ± 58.63), Zn (394.03 ± 95.40), Mo (2.65 ± 0.41), Fe (562.87 ± 860.35), Si (5728.20 ± 2946.46), Co (2.11 ± 0.85), Cd (0.38 ± 0.37), and Ce (7.92 ± 5.66). The EC and pH (mean ± SD) of sand suspension (1:5 sand to distilled water) was 5.88 ± 0.28 µScm^-1^ and 7.40 ± 0.10, respectively. SEM revealed that the sand had a crystal-like structure of grains, and EDX confirmed the presence of elements such as Si, Al, and O (**Figures 1A, C**). SEM images confirmed that nanoparticles do exist in the company given estimated diameter (**Figures 1B, D**) and EDX revealed the presence of elements such as Ce, O, and Cl in NPs.

### 3.2 Maize seedling biomass, height, and diameter

**Figure 2:**
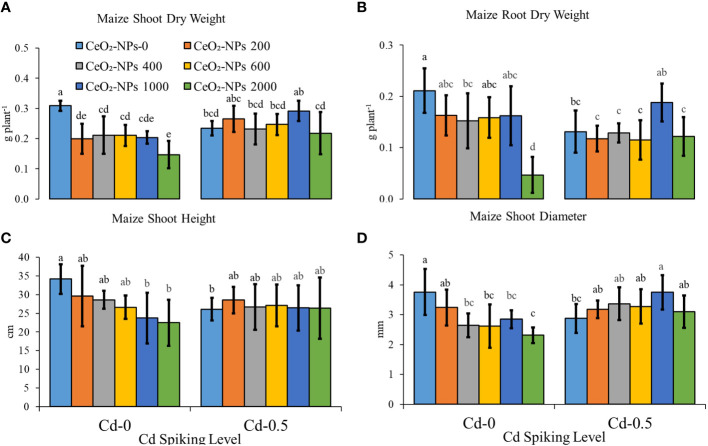
Effect of CeO_2_-NPs applied at 200, 400, 600, 1000 and 2000 mg kg^-1^ dry sand, alone and in combination with 0.5 mg kg^-1^ Cd on maize growth. **(A)** shoot dry weight (g), **(B)** root dry weight (g), **(C)** plant height (cm), **(D)** plant diameter (mm). The graphs are of mean (*n* = 4) ± standard deviation. Letters shown pair wise comparison via Least Significant Difference (LSD) at *p* ≤ 0.05.

CeO_2_-10 nm NPs at maximum dose (2,000 mg kg^-1^) showed net toxicity with regard to maize seedling shoot and root biomass, with a significant decrease of 52.51% and 77.89%, respectively, compared with the uncontaminated control (UCC). Additionally, Cd (0.5 mg kg^-1^) application resulted in a net toxicity with regard to seedling shoot and root dry weights. When NPs were applied in combination with Cd (0.5 mg kg^-1^), a net ameliorative effect on shoot and root dry weights was observed (a respective net increment of 24.28% and 43.42% compared with the respective CC); however, compared with the CC, this effect was non-significant. The 2,000 mg kg^-1^ NP dose, which had a net toxic effect (29.37% and 42.18% decrease in shoot and root fresh weight compared with the UCC) when applied alone, showed a net increase in seedling shoot and root growth (compared with only 2,000 mg kg^-1^ NPs applied sets) when applied along with Cd, suggesting a somewhat ameliorative effect as well. Maize seedling height and diameter were also found to be decreasing with Cd toxicity and CeO_2_-NPs applied alone or in combination with Cd, a net increment in both parameters was observed (significant for diameter only) (**Figure 2**).

### 3.3 Maize root growth parameters

**Figure 3:**
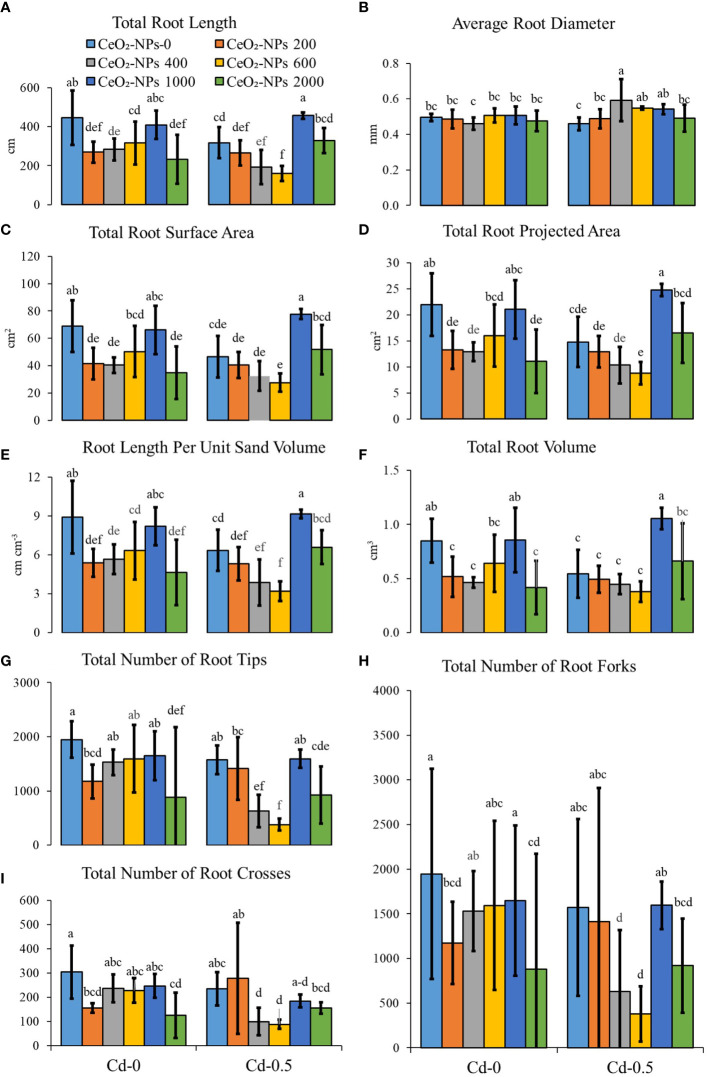
Effect of CeO_2_-NPs applied at 200, 400, 600, 1000 and 2000 mg kg^-1^ dry sand alone and in combination with 0.5 mg kg^-1^ Cd on maize root growth. **(A)** total root length (cm), **(B)** average root diameter (mm), **(C)** root length per unit volume (cm per cm^3^ soil), **(D)** number of root tips, **(E)** number of root forks, **(F)** number of root crosses, **(G)** total rot surface area (cm²), **(H)** total root projected area (cm^3^), **(I)** total root volume (cm^3^). The graphs are of mean (*n* = 4) ± standard deviation. Letters shown pair wise comparison via Least Significant Difference (LSD) at *p* <0.05.

The application of CeO_2_-10-nm NPs had a significant effect on root growth parameters for both the Cd-0- and Cd-spiked sets but there was a divergence in effects. The UCC had maximum root length, which decreased with CeO_2_ NPs applied alone, and Cd stress also caused an overall decrease in root length. With the co-application of CeO_2_-10-nm NPs and Cd, the toxicity was enhanced with 200−600 mg kg^-1^ of NPs and 0.5 mg kg^-1^ of Cd, whereas 1,000 mg kg^-1^ of NPs resulted in toxicity reversal with maximum root length. The average root diameter was not affected by the application of CeO_2_-NPs or Cd (alone), whereas in combination, CeO_2_-NP application at 400, 600, and 1,000 mg kg^-1^ resulted in a higher net root diameter compared with the contaminated control (CC). Additionally, root length per unit volume decreased with the introduction of CeO_2_-NPs in normal sand pots, while a net increment in toxicity was observed (as observed in root length) when NPs were applied with Cd, except with a 1,000 mg kg^-1^ NP dose, suggesting the efficacy of NPs at reversing toxicity, which might be due to the lack of an effect of Cd adsorption in maize shoots (discussed below). Furthermore, the number of tips, forks, and crosses were at the maximum in the UCC and a net decrease was observed with the introduction of Cd. The application of NPs alone resulted in no significant increment in any of these parameters. The application of Cd with NPs at 400 and 600 mg kg^-1^ resulted in enhanced toxicity. Total root surface area was significantly affected by the application CeO_2_-NPs in Cd-0 sets, with visible toxicity in the 200-, 400-, and 2,000-mg kg^-1^-spiked pots; in Cd-0.5 sets, this toxicity was still visible but only with a 1,000 mg kg^-1^ dose of NPs (in which a significant increase of 67.41% was observed compared with the CC). A similar trend was observed in total root projected area, in which a 1,000 mg kg^-1^ dose of CeO_2_-NPs only resulted in net higher values compared with the CC in the Cd-0.5 set. The total root volume of maize seedlings spiked with 200, 400, 600, and 2,000 mg of CeO_2_-NPs per kg of sand was at a minimum for the Cd-0 set, while no significant toxicity with 1,000 mg kg^-1^ was observed for Cd-0.5, resulting in a net 94.02% increment in root volume compared with the CC, as shown in **Figure 3**.

### 3.4 Shoot and root cerium and cadmium distribution

**Figure 4:**
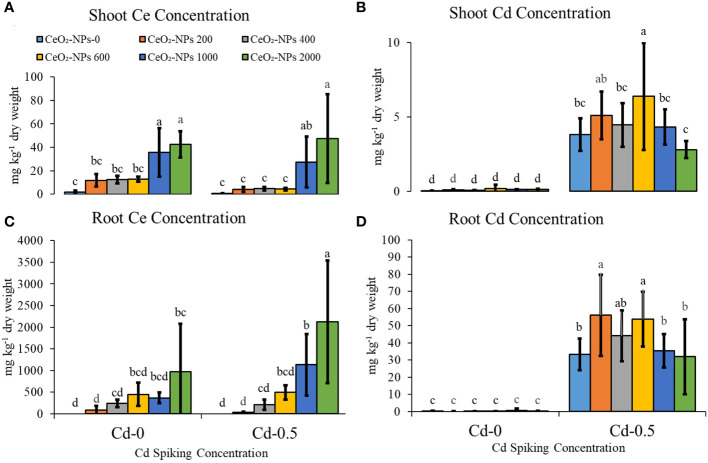
Effect of CeO_2_,-NPs applied at 200, 400, 600, 1000 and 2000 mg kg^-1^ dry sand alone and in combination with 0.5 mg kg^-1^ Cd on maize Cd and Ce contents. **(A)** shoot ce (mg kg dry weight), **(B)** root ce (mg kg^-1^ dry weight), **(C)** shoot cd (mg kg^-1^ dry weight), **(D)** root cd (mg kg^-1^ dry weight). The graphs are of mean (*n* = 4) ± standard deviation. Letters shown pair wise comparison via Least Significant Difference (LSD) at *p* ≤ 0.05.

NP application resulted in a significantly higher Ce uptake in the shoots of maize seedlings, although NP doses of 200, 400, and 600 mg kg^-1^ did not result in a net difference in shoot Ce content in both the Cd-0 and Cd-0.5 sets. Although a higher uptake was observed with 1,000- and 2,000-mg kg^-1^-supplemented pot sets, these two treatments did not result in significant variation in either set (Cd-0 and Cd-0.5). For root Ce content, maximum concentration was observed in Cd-spiked 2,000-mg kg^-1^-supplemented sets, with a total value of 2,124 ± 1,414 (mean concentration in mg per kg dry mass ± standard deviation). Cd contamination in combination with 2,000 mg kg^-1^ of CeO_2_-NPs resulted in higher Ce uptake in roots compared with NPs alone application, suggesting that seedlings had a preference for combined stress. For shoot Cd, doses of 200 or 600 mg kg^-1^ of CeO_2_-NPs resulted in higher Cd accumulation in shoots compared with the control, and only a 2,000 mg kg^-1^ dose of NPs resulted in a net decrease in shoot Cd. However, the difference was not significant compared with the control. For root Cd content, doses of 200, 400, or 600 mg kg^-1^ of CeO_2_-NPs resulted in higher root Cd content compared with the control, but higher NP doses (1,000 and 2,000 mg kg^-1^) had no significant effect **Figure 4**.

### 3.5 Shoot and root micronutrients and beneficial elements

**Figure 5:**
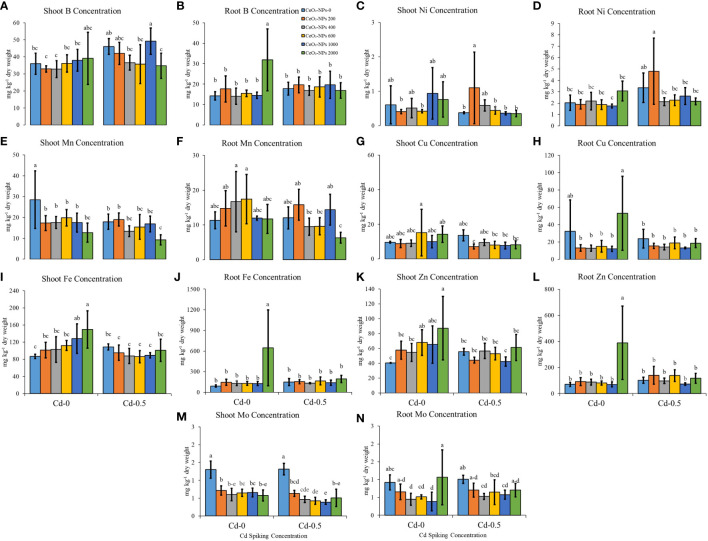
Effect of CeO_2_-NPs applied at 200, 400, 600, 1000 and 2000 mg kg^-1^ dry sand alone and in combination with 0.5 mg kg^-1^ Cd on maize Cd and Ce contents. **(A)** shoot b (mg kg^-1^ dry weight), **(B)** root b (mg kg^-1^ dry weight); **(C)** shoot ni (mg kg^-1^ dry weight), **(D)** root ni (mg kg^-1^ dry weight); **(E)** shoot mn (mg kg^-1^ dry weight), **(F)** root mn (mg kg^-1^ dry weight); **(G)** shoot cu (mg kg^-1^ dry weight), **(H)** root cu (mg kg^-1^ dry weight); **(I)** shoot fe (mg kg^-1^ dry weight), **(J)** root fe (mg kg^-1^ dry weight); **(K)** shoot zn (mg kg^-1^ dry weight). **(L)** root zn (mg kg^-1^ dry weight); **(M)** shoot mm (mg kg^-1^ dry weight), **(N)** root mn (mg kg^-1^ dry weight). The graphs are of mean (*n* = 4) ± standard deviation. Letters shown pair wise comparison via Least Significant Difference (LSD) at *p* ≤ 0.05.

Variable CeO_2_-NP doses have shown divergent effects on some micronutrients, which also differs from element to element. For shoot B content (mg per kg dry weight), the highest values were observed in Ce1000+Cd-spiked sets (49.20 ± 7.77; mean mg kg^-1^ dry weight ± standard deviation), while in roots, a 2,000 mg kg^-1^ dose of CeO_2_-NPs alone resulted in the highest B content (31.87 ± 15.13). Shoot Mn content was highest for the control (UCC), while the lowest content was observed in the Ce2000+Cd-spiked sets (9.27 ± 2.47). Similarly, for root Mn content, Ce2000+Cd-spiked sets resulted in the lowest concentration (6.31 ± 1.56). In non-Cd-spiked sets, NPs caused a gradual increase in shoot Fe content, with the highest content (149.72 ± 43.56) observed in the 2,000-mg kg^-1^ CeO_2_-NPs sets; no significant difference was observed in the Cd-spiked sets. For root Fe content, again 2,000 mg kg^-1^ of CeO_2_-NPs alone resulted in the maximum Fe content (645.29 ± 551.28), while other treatments varied non-significantly, with the combination of Cd and 2,000 mg kg^-1^ of CeO_2_-NPs lowering Fe accumulation (193.65 ± 52.59), suggesting the accumulation of Fe was hindered in this instance. NPs applied alone (without Cd spiking) resulted in no significant changes in shoot and root Ni content, while in Cd-spiked sets, 200 mg kg^-1^ CeO_2_-NPs resulted in the highest shoot (1.1 ± 1.04) and root Ni (4.80 ± 2.90) content. The application of CeO_2_-NPs had no significant effect on shoot and root Cu content; however, in Cd-0 sets, seedling Zn was highest in the 2,000-mg CeO_2_-NPs per kg sand sets with the highest shoot (87.26 ± 42.72) and root (388.80 ± 280.68) Zn content. The other treatments did not significantly affect tissue Zn content. Shoot Mo content was highest in the UCC (1.30 ± 0.24) and the CC (1.31 ± 0.16), and the application of NPs resulted in significant decreases in shoot Mo content. For root Mo, variable effects of NPs were observed (**Figure 5**).

**Figure 6:**
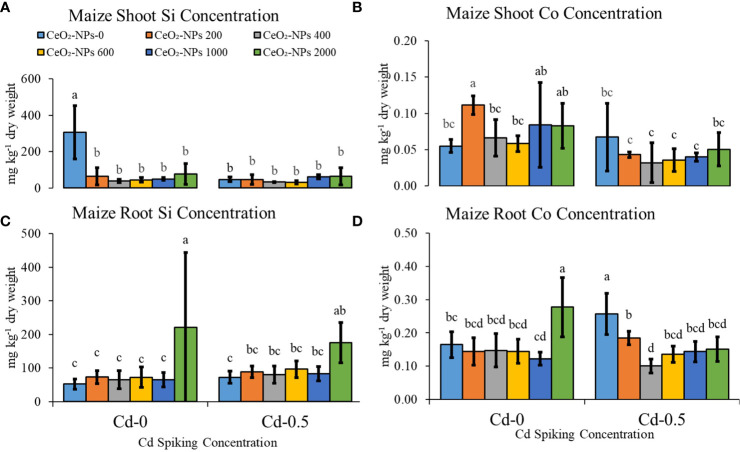
Effect of CeO_2_-NPs applied at 200, 400, 600, 1000 and 2000 mg kg^-1^ dry sand alone and in combination with 0.5 mg kg^-1^ Cd on maize Co and Si contents. **(A)** Shoot Si (mg kg^-1^ dry weight), **(B)** Shoot Co (mg kg^-1^ dry weight), **(C)** Root Si (mg kg^-1^ dry weight). **(D)** Root Co (mg kg^-1^ dry weight). The graphs are of mean (*n* = 4) ± standard deviation. Letters shown pair wise comparison via Least Significant Difference (LSD) at *p* < 0.05.

Si and Co, important beneficial elements for plants, were not applied exogenously but were found in plant tissues due to the potential of sand to provide significant amounts of both nutrients (described in the Material Characterization section). Shoot Si content was highest in the control (CC), while for roots, the highest content was observed in Cd-0 and Cd-0.5 sets supplemented with 2,000 mg CeO_2_-NPs per kg sand. For shoot Co, in Cd-0 sets, 200 mg kg^-1^ of CeO_2_-NPs resulted in a higher content, while for roots, Co content was highest in the 2,000 mg kg^-1^ CeO_2_-NP-spiked sets. In Cd-0.5 sets, shoot Co did not vary significantly, while for root Co, net decreases were observed when NPs were added (**Figure 6**).

### 3.6 Root apoplastic barriers

**Figure 7:**
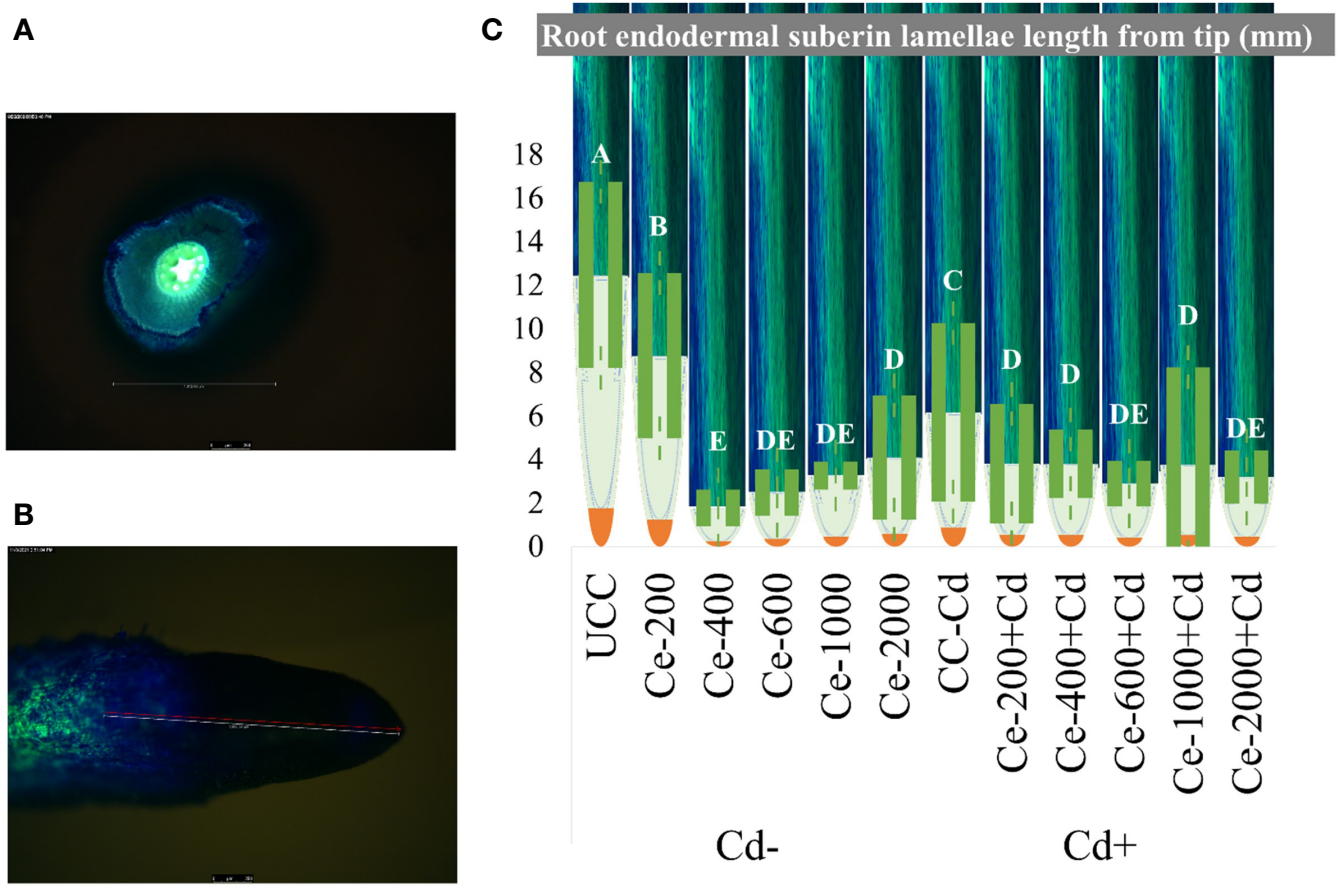
Root endodermal Suberin Lamellae (barrier formation) length on maize seedling roots under CeO2-NPs applied at 200, 400, 600, 1000 and 2000 mg kg^-1^ dry sand alone (Cd-) and in combination with 0.5 mg kg^-1^ Cd (Cd+). **(A)** Cross section of root with full barrier formation, **(B)** The gradual development of barriers from tip (green color development), **(C)** Mean root barrier distance from root tip. The error bars given on mean are of standard deviation (SD).

Root apoplastic barrier formation was visualized using florescence microscopy, and CeO_2_-NPs significantly affected the development of root barriers (recorded as the length from the tip). Cd stress (0.5 mg kg^-1^ of sand) resulted in a net decrease of barrier length from the tip, which maize seedlings typically do to control the uptake of pollutants. The nanoparticles alone and in combination with Cd further reduced the length of the root barrier from the tip, as presented in **Figure 7C**. Fully developed root barriers are shown in **Figure 7A**, presented in a cross-section of a root, while categorical length measurement from the tip and a visual representation of barrier formation (in green) is shown in **Figure 7B, C**.

## 4 Discussion

The application of CeO_2_-NPs through difference sources and concentrations has some beneficial effects on plants (Cao et al., 2017; Fox et al., 2020; Lizzi et al., 2021), but the generalized effects cannot be defined at once due to the divergence in the effects of bulk and ionic parts of cerium (Zhang et al., 2015; Barrios et al., 2016; Lizzi et al., 2021). The size of CeO_2_-NPs can limit their entry into plant cells as membrane pore size can reduce the translocation of NPs *via* cross root epidermal cell membranes (Ma et al., 2010a; Ma et al., 2010b; Rico et al., 2011), where these NPs tend to adopt apoplastic pathways. Within this pathway, the translocation of NPs is controlled by various barriers, such as Casparian strips (Roppolo et al., 2011), through which they can reach the xylem and accumulate in the upper parts of the shoots (Lv et al., 2019). NPs larger than 20 nm cannot pass through the cell wall pores easily, while cuticular pathways can allow the uptake of solutes that are 0.6−4.8 nm in size (Popp et al., 2005, Eichert et al., 2008, Eichert and Goldbach, 2008). The uptake of CeO_2_-NPs smaller than 20 nm might be higher in plants, which when coupled with surface property variation may potentially increase their toxicity (Cresi et al., 2017). CeO_2_-NPs smaller than 10 nm can alter a plant’s physiology (Corral-Diaz et al., 2014) and growth parameters positively (Tassi et al., 2017) as well as negatively at higher concentrations, as observed for rice (Rico et al., 2013a; Rico et al., 2013b) and maize (Zhao et al., 2012). The toxicity of very fine Ce NPs can be due to their effect on plant morphology, nutritional acquisition, antioxidant defense, or molecular processes (Prakash et al., 2021).

In the present investigation, the effects of 10-nm CeO_2_-NPs on plant growth were negative overall, with the highest toxicity observed with the highest applied concentration (2,000 mg kg^-1^); however, when applied in combination with Cd (0.5 mg kg^-1^), the NPs tended to nullify Cd toxicity, with a dose of 1,000 mg kg^-1^ showing the most benefit at all levels. CeO_2_-NPs at 200 mg kg^-1^ of cerium concentration decreased the uptake of heavy metals (Cu, Mn, Zn, and Fe) in *Pisum sativum* and reduced the height of *Solanum lycopersicum* L.; no effect was observed on biomass (Skiba and Wolf, 2019). Similarly, CeO_2_-NPs at 0.1–10 mg/L^−1^ in *Solanum lycopersicum* L have shown no significant effect at lower concentrations, while at higher concentrations they have exhibited toxicity with regard to plant biomass (Wang et al., 2012). Additionally, the application of CeO2-NPs alone decreased plant root growth parameters, with the highest toxicity observed in 2,000 mg kg^-1^-spiked sets. When applied in combination with Cd, 1,000 mg kg^-1^ of CeO2-NPs caused a net reversal in toxicity and positively affected maize seedlings to a degree.

CeO_2_-NPs can modify plant physiology and nutritional aspects, as reported in *Triticum aestivum* L. (Rico et al., 2014), but these effects are subject to the potting medium, source, and NP dose, as these NPs have been shown to be toxic in plants (Ayub et al., 2023). The Ce and Cd content of plant tissues were in agreement with the applied concentrations and NPs have shown some effect in controlling shoot Cd content, with lower concentrations increasing the net translocation of Cd into shoots and higher concentrations (2,000 mg kg^-1^) somewhat decreasing shoot Cd content. The increasing shoot Cd content with 200, 400, and 600 mg kg^-1^ NP doses can explain the overall decreased root dry weight, while higher NP doses caused a reversal *via* decreasing Cd uptake (although the effect was not significant). The main mechanisms involved could be specific adsorption (Fouda-Mbanga et al., 2022) and the development of root barriers to counteract Cd translocation into plant bodies (Rossi et al., 2016; Rossi et al., 2017; Rossi et al., 2018), which is highly dependent on NP size (Ayub et al., 2023). The tested concentrations of CeO_2_-NPs controlled the uptake and translocation of micronutrients and beneficial elements (Si and Co). The concentrations of the micronutrients Fe, Zn, and B increased in maize seedling shoots with the application of CeO_2_-NPs alone; however, in combination with Cd (Cd-0.5), no significant effect was observed. In a study conducted by Pošćić et al. (2016), it was evident that cerium NPs and, in particular, titanium oxide NPs (applied at 500 and 1,000 mg kg^-1^) altered amino acid, crude protein, macronutrient, and micronutrient content in barley. Skiba and Wolf (2019) suggested that CeO_2_-NPs applied at 200 ppm of Ce can decrease Cu, Zn, and Fe uptake, but in our case, NPs increased shoot Fe and Zn content, while shoot Mn content decreased. When NPs were applied in combination with Cd, variable effects were observed.

**Figure 8:**
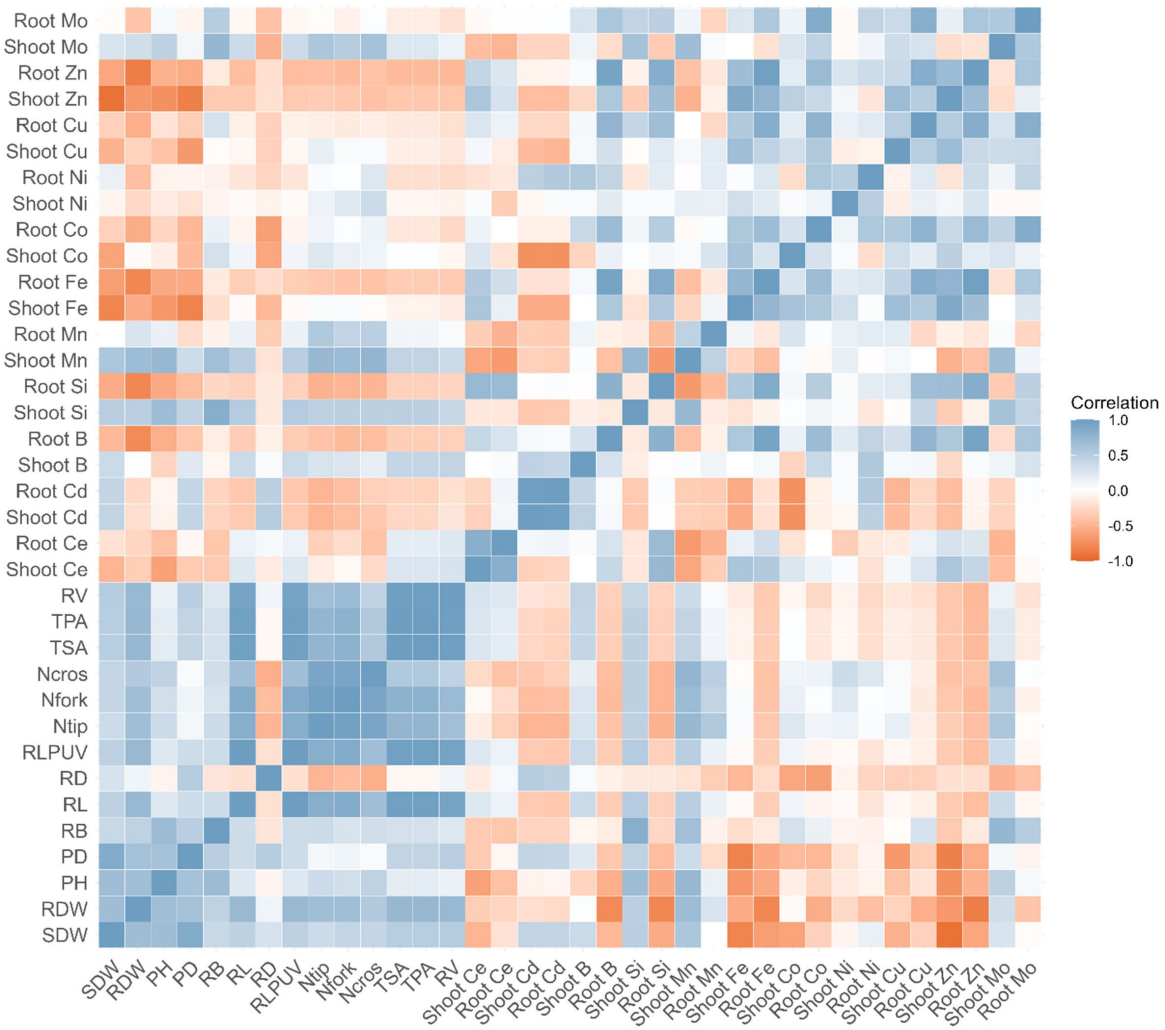
The Correlation tree map among acquired parameters of maize seedling (*n* = 12) as, shoot dry weight (SDW), root dry weight (RDW), plant height (PH), plant diameter (PD), root barrier length (RB), total root length (RL), average root diameter (RD), root length per unit volume (RLPUV), number of root tips (Ntip), number of root forks (Nfork), number of root crosses (Neros), total root surface area (TSA), total root projected area (TPA), total root volume (TV). shoot and root elemental contents (Ce, Cd, B, Si, Mn, Fe, Co, Ni, Cu, Zn, Mo).

The various studies on CeO_2_-NPs in plants were conducted using concentrations of 1−1,000 mg/L^-1^ (Wang et al., 2012; Holden et al., 2014; Rossi et al., 2017; Skiba and Wolf, 2019), and for toxicity studies, a dose of 2,000 mg/L^-1^ (Zhang et al., 2019) was used; however, the present investigation was conducted using dry sand, and NPs were applied on the basis of weight. The findings of this study conclude that CeO_2_-10 nm NPs are significantly toxic to plants when applied alone, although they can help reverse Cd toxicity to a certain extent. The correlation tree map shows that the heavy metal nutrients Zn, Cu, and Ni in shoots were negatively correlated with shoot growth parameters, suggesting the uptake of these nutrients was above permissible limits, and shoot Ce was also negatively correlated with shoot growth, suggesting significant effects when applied from nano sources. Root growth parameters were positively correlated with plant shoot growth, showing that healthy roots can affect plant shoot growth and development (**Figure 8**).

## 5 Conclusion

CeO_2_-10 nm NPs were toxic for corn growth when applied alone, while in combination with Cd, no significant toxicity on corn growth was observed as the 1,000 mg kg^-1^ dose increased shoot growth. The effect on root growth was variable due to the divergent uptake of nutrients, beneficial elements, Ce, and Cd. The NPs also altered root barrier formation and showed that they could potentially affect plant root anatomy.

## Data availability statement

The original contributions presented in the study are included in the article/Supplementary Material. Further inquiries can be directed to the corresponding author.

## Author contributions

MA: conceptualization, methodology, formal data analysis, investigation, funding acquisition, writing-original draft, and data visualization. MR: conceptualization and project administration. HA: conceptualization and project administration. CR: methodology, investigation, resources, writing-review, and editing. GA: Proofreading, review, and editing. WU: statistical analysis, proofreading, review, and editing. AW: methodology, investigation, resources, writing-review, and editing. MN: writing-original draft and proofreading. J-PF: methodology, writing-review, and editing. LR: conceptualization, supervision, methodology, resources, writing-review, editing, and project administration. All authors contributed to the article and approved the submitted version.
